# A Novel Dynamic Bit Rate Analysis Technique for Adaptive Video Streaming over HTTP Support

**DOI:** 10.3390/s22239307

**Published:** 2022-11-29

**Authors:** Ponnai Manogaran Ashok Kumar, Lakshmi Narayanan Arun Raj, B. Jyothi, Naglaa F. Soliman, Mohit Bajaj, Walid El-Shafai

**Affiliations:** 1 Department of Computer Science Engineering, Koneru Lakshmaiah Education Foundation, Vaddeswaram 522 302, India; 2 Department of Computing Science and Engineering, B.S.A. Crescent Institute of Science and Technology, Vandalur, Chennai 600 048, India; 3 Department of Electrical and Electronics Engineering, Koneru Lakshmaiah Educational Foundation, Vaddeswaram 522 302, India; 4 Department of Information Technology, College of Computer and Information Sciences, Princess Nourah bint Abdulrahman University, P.O. Box 84428, Riyadh 11671, Saudi Arabia; 5 Department of Electrical Engineering, Graphic Era (Deemed to Be University), Dehradun 248 002, India; 6 Security Engineering Lab, Computer Science Department, Prince Sultan University, Riyadh 11586, Saudi Arabia; 7 Department of Electronics and Electrical Communications Engineering, Faculty of Electronic Engineering, Menoufia University, Menouf 32952, Egypt

**Keywords:** adaptive video streaming, A-LSTM networks, bit rate measurement, client–server model, HTTP, reference metrics, video quality

## Abstract

Recently, there has been an increase in research interest in the seamless streaming of video on top of Hypertext Transfer Protocol (HTTP) in cellular networks (3G/4G). The main challenges involved are the variation in available bit rates on the Internet caused by resource sharing and the dynamic nature of wireless communication channels. State-of-the-art techniques, such as Dynamic Adaptive Streaming over HTTP (DASH), support the streaming of stored video, but they suffer from the challenge of live video content due to fluctuating bit rate in the network. In this work, a novel dynamic bit rate analysis technique is proposed to model client–server architecture using attention-based long short-term memory (A-LSTM) networks for solving the problem of smooth video streaming over HTTP networks. The proposed client system analyzes the bit rate dynamically, and a status report is sent to the server to adjust the ongoing session parameter. The server assesses the dynamics of the bit rate on the fly and calculates the status for each video sequence. The bit rate and buffer length are given as sequential inputs to LSTM to produce feature vectors. These feature vectors are given different weights to produce updated feature vectors. These updated feature vectors are given to multi-layer feed forward neural networks to predict six output class labels (144p, 240p, 360p, 480p, 720p, and 1080p). Finally, the proposed A-LSTM work is evaluated in real-time using a code division multiple access evolution-data optimized network (CDMA20001xEVDO Rev-A) with the help of an Internet dongle. Furthermore, the performance is analyzed with the full reference quality metric of streaming video to validate our proposed work. Experimental results also show an average improvement of 37.53% in peak signal-to-noise ratio (PSNR) and 5.7% in structural similarity (SSIM) index over the commonly used buffer-filling technique during the live streaming of video.

## 1. Introduction

Adaptive media streaming through Hypertext Transfer Protocol (HTTP) is a widely used mechanism by the service provider. The main advantage is that it does not require any change in the underlying network layer to support streaming. The standard organization Moving Picture Experts Group (MPEG) and the 3rd Generation Partnership Project (3GPP) have standardized a method called Dynamic Adaptive Streaming over HTTP (DASH) to ensure interoperability [1]. In DASH implementation, the video is segmented, and each segment is stored with different video quality parameters, including spatial and temporal resolutions. The adaptation process at the server streams suitable segments targeted to match the link capacity of the client [2]. The present solution fails in case of fast changes in network bit carrying capacities leading to the video freezing and enhancing degradation of user satisfaction [3]. A new method can be integrated with the DASH technique to support live content streaming. In the buffer-based implementation of live video streaming [4,5], involving client observation of buffer threshold does not guarantee the quality since the variation in the bit rate depends on capturing and coding methods at the server. The scalable video coding (SVC) approach permits frame-level adaptation, but it requires switching different video layers during the session [6]. 

**Figure 1:**
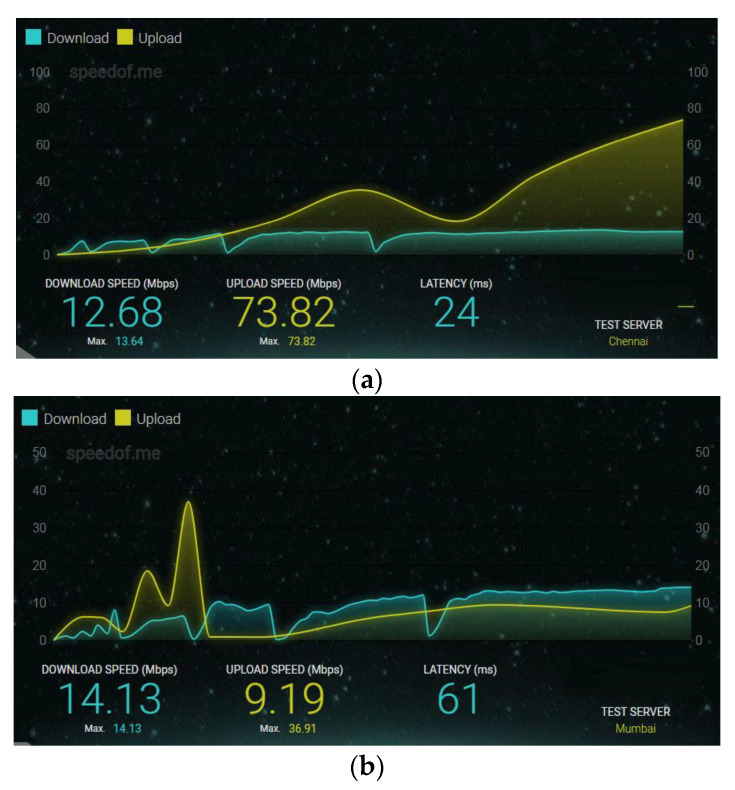
Download/Upload bit rate observed through different wireless Internet dongles (**a**) Reliance Netconnect+ (CDMA20001xEVDO Rev-A) 4G dongle (**b**) Airtel 4G Mobile Hotspot (LTE-TDD Category 3) dongle.

Another development in adaptive video streaming is the study of 3G/4G cellular networks offering Internet connection. Many times, the user equipment offered by a cellular operator to support Internet services fails to deliver the desired quality for many practical reasons. For example, Figure 1 shows the observation of a 4G dongle employing the CDMA20001xEVDO Rev-A technique and a 4G dongle based on the long-term evolution time-division duplex (LTE-TDD) category-3 system. The observed variation in bit rate depends on location and fluctuation with time. This clearly justifies the motivation behind the developing system, which can target wireless empowerment and offer the best performance in terms of user satisfaction.

The objective performance of the proposed system is evaluated using standard metrics while meeting the design goal. The International Telecommunication Union Standardization Sector (ITU-T) recommends using full reference metrics when the original video is available at the receiver to test the individual system in a laboratory environment. The standard evaluation metrics can be applied to test video quality in different formats, including quarter common intermediate format (QCIF), common intermediate format (CIF), and Video Graphics Array (VGA). Table 1 lists the different parameters and corresponding values to test the large varying quality of the video. 

The proposed work in this paper tries to use these parameters with their corresponding standard values in implementation and development. The existing link bit rate assessment method at the client involves sending a ping message to the server and computing the bit rate using the time spent by the packet to come back. However, this method lacks precision because of many external factors, for example, instantaneous congestion to the router can temporarily interrupt the incoming rate of a ping message. Thus, the best practice for dealing with this issue is to evaluate the capacity of the link in terms of bit rate to the receiver by analyzing the bit stream arrival on the fly.

**Figure 2:**
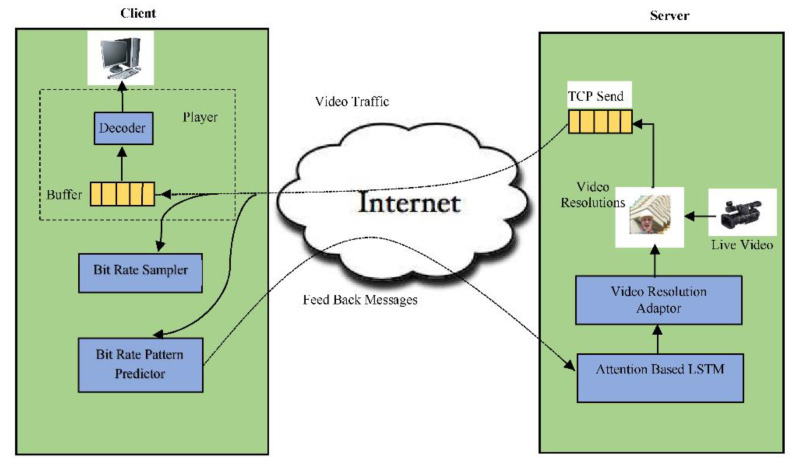
Schematic diagram of a client–server model for adaptive video streaming.

The streaming that needs to be sampled at times is analyzed and sent a feedback message to the sender for performing remedial action on the outgoing stream so that the end user enjoys a better quality of experience during the entire viewing session. The schematic approach of the proposed architecture is shown in Figure 2, where the client applies a predefined algorithm to compute the arrival rate and forward the report to the server. The response action in the system loop needs to be proactive and stable to meet the satisfaction of the system’s real-time streaming requirement. This provides the scope of additional intelligence for the link capacity estimation. In the proposed method, pattern matching is employed by the client to reduce the processing time and meet the requirements of live video streaming.

An analytical model is included to support the performance measure of the proposed work. The bit rate profile and performance measure are also presented in tabular form. Although the proposed system is basically developed as on-the-top of HTTP (OTT), it has incorporated the inherent behavior of streamed data over the wireless network. The dynamics of the observed bit rate are due to the burst nature of the Internet traffic [7] and the time-varying nature of wireless signals in 3G/4G networks. Further, the system performance evaluated here corresponds to the Internet over 4G wireless networks (CDMA20001xEVDO Rev-A). Finally, the proposed work is compared against popular buffer-filling methods [8], the default Internet option to stream multimedia content.

We present a summary of our contributions:

1. We devised a novel feed-forward attention-based LSTM model using reinforcement learning to successfully integrate features from several layers of the LSTM network to solve sequential bit rate dependency problems and adaptive video streaming over HTTP.
2. We present a cost function for attention networks that maximizes video quality and minimizes re-buffering time.
3. Experimental results have revealed that the suggested A-LSTM technique performs better than the state-of-the-art buffer filling algorithms on the standard datasets.

The rest of the paper is organized as follows: video streaming, video buffering, and machine learning techniques in multimedia streaming-related works are summarized in Section 2. The architecture of the client–server model and A-LSTM is introduced first. Then, we present the suggested A-LSTM model employing reinforcement learning and feed forward attention-LSTM technique in Section 3. Section 4 presents the results of tests performed on standard datasets containing variant bit rates and buffer lengths with the current popular techniques. Lastly, we complete the paper with the hypotheses and future work in Section 5.

## 2. Related Work

The bit rate adaptation of video streaming involves many factors, including scheduling of segmented video, bit rate selection, bandwidth estimation, etc. Many commercially available services, such as Smooth Streaming, Akamai HD, Netflix, and Adobe OSMF, implement adaptive streaming through the Internet. Appropriate modeling and analysis of the key phase include switching of the multimedia data during streaming used by the service provider, which helps to refine the design of the system for improving performance in the feedback control loop [9].

In the HTTP based adaptive streaming (HAS), the quality of experience (QoE) depends on the appropriate selection of video segment and switching of bit stream based on client input [10]. The underflow probability of the media buffer is estimated during run time and is incorporated in the QoE framework while supporting the acceptable quality of the streaming video. The buffer stability is a vital parameter to maintain the quality of the video during play out, and this is implemented by estimating the buffer level during the streaming session of the client [11]. Furthermore, the estimation of buffer underflow probability can provide vital inputs in implementing layer switching of different video segments, i.e., adaptation of video content during the streaming process [12]. 

A new version of adaptive rate control algorithms [13] is proposed to improve the combined system performance of video play out smoothness and frame quality based on the feedback information of wireless network estimation, buffer content, and playback situation. However, their main disadvantage is the lack of adaptability of heterogeneous networks and noisy error data. To solve transmission errors, a novel error control coding technique is proposed [14] for video transmission over wireless network and to implement different error control techniques for video transmission. However, their main performance is not evaluated for real-time applications and does not consider the pixel intensity values.

To solve the pixel intensity problem, a novel algorithm [15] is presented for exploiting a general model of high-efficiency video coding (HEVC) technique with the help of decoding-energy fast compression (DEFC). This method does not consider routing parameters. A Novel Analytical framework [16] is proposed based on routing measure parameters to reduce distortion in wireless video traffic. A new hue saturation lightness (HSV), edge preserving, and Huffman-coding (HC)-based Huffman and differential pulse code modulation (DPCM) encodings algorithm [17] is proposed to increase the compression ratio of the video frames.

Dynamic Adaptive Streaming over HTTP (DASH) in the client–server environment has attracted worldwide attention for many reasons from researchers and developers [18]. There is a need to map the DASH layer with the scalable video coding (SVC) layer, not only to improve the throughput of streaming video with the help of HTTP overhead messages, but also to estimate the bit rates of media sessions [19]. A cross-layer method involving DASH and a physical (radio) layer can manage better scheduling and resource allocation [20] in the media accesses control layer to solve the throughput problem. To overcome the limitations of a single network, energy consumption of the end device and environmental factors are considered an important parameter by [21] to seamlessly transfer the requested video segments concurrently to mobile devices. 

Further, a learning approach may help with streaming video through multilink. For example, the learning method can incorporate a Markov decision process with a finite state. The reward calculation in such implementations must include video quality of service (QoS) [21,22]. The estimation of network bandwidth as a Transport Control Protocol (TCP) throughput in the HAS system by the client may not be reliable when HAS traffic occupies a significant portion of the network traffic. The client encounters bottlenecks in the networks for supporting discrete characteristics of video bit rate while competing with other clients [23]. The physical layer information, i.e., statistics at the modem, can be passed to the application layer for fast identification and estimation of the wireless channel condition in a HAS system [24]. In [25], the physical layer throughput/goodput is used to adapt the rate of the HAS video client and improve the QoE of streaming video, but still, the system needs to consider the dynamic behavior of the wireless channel. At the physical layer, modem statistics can detect sharp variations in wireless link quality. Now, the HAS client can place a new request to the server based on its current state and the status of the existing request for the segment (as shown in Figure 2).

Another focus for adaptation of DASH/HAS video in cellular mobile systems can be enforced by the network operator based on the knowledge of cell load and channel conditions to optimize the content delivery. Further, this opens an avenue for joint optimization of resource allocation in multi-user networks and controlling video streaming for the DASH client [26]. Furthermore, assuming the proxy has the adaptive HAS content with multiple-bit rate encoding, it may eliminate the need for further processing of video, and this approach is desirable for on-the-top (OTT) streaming services, particularly when the DASH server is not present in the network operator’s domain.

Understanding the time complexities and other quality of service (QoS) requirements of live video streaming, our work considers sampling of incoming bit rate alone and the buffer state for the client’s decision-making. Furthermore, the proposed system is developed to cope with the fluctuation in bit rate due to the best-effort model of the Internet and the time-varying characteristics of a wireless channel. The combined network effect on the sampled data set (bit rate) tends to behave as random variables. Hence the Markov process-based decision-making is not suitable here [26]. Another novel contribution compared to the earlier work is the quick processing of link capacity estimation by using predefined pattern matching, as the system design is targeted to handle live video streams.

## 3. Proposed Methodology

### 3.1. System Architecture

**Figure 3:**
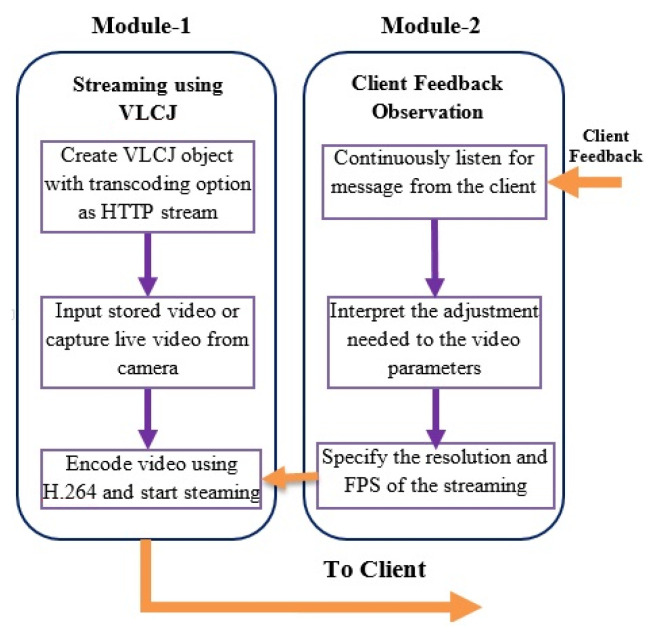
Modular flow diagram at server side representing transcoding, client feedback analysis, and adaptive video streaming operations.

**Figure 4:**
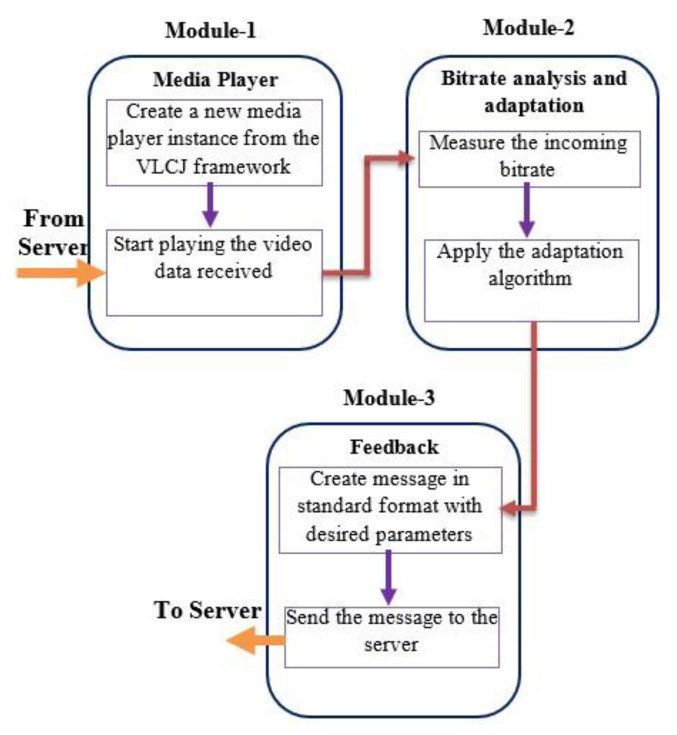
Modular flow diagram at client side performing bit rate analysis and giving feedback to the server.

The proposed system is demonstrated after the client–server architecture. The main function of the server side module is to receive the live or stored video for transcoding before streaming (Figure 2). The system architecture consists of two main modules, where the first module deals with transcoding and adaptive streaming of the content while the second module listens to the client feedback. A video-acquiring device capable of capturing high-definition content is attached to the server (Figure 3), and the H.264 video codec codes the resulting stream. The connection for live streaming is implemented on 4G wireless cellular networks. The system implementation at the client deals with (Figure 4) playing and analysis of the incoming video stream. After collecting N frames, the system simultaneously calls the bit rate estimation process and the media player task. The bit rate estimation algorithm defines the feedback category to be sent to the server.

**Figure 5:**
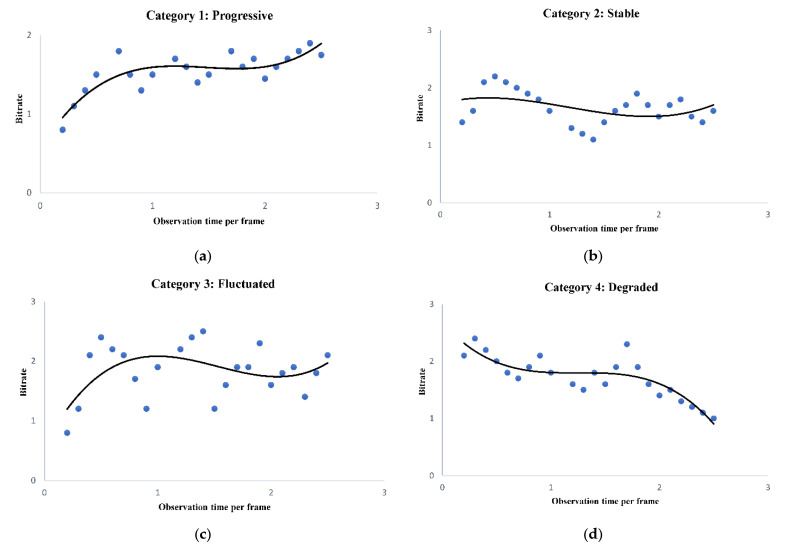
Different categories of bit rate arrived at the client side. (**a**) Progressive data pattern. (**b**) Fluctuated data pattern, (**c**) Stable data pattern, (**d**) Degraded data pattern.

The four unique patterns of variation in bit rate are defined (Figure 5) for comparison and analysis of streaming data. Category 1 denotes a progressive type of data flow where the bit rate increases in time. The bit rate fluctuation may gradually cease and finally stabilize (Category 2). Category 3 represents a case when the variation in bit rate diverges. If the decreasing trend of the bit rate continues, it represents a disturbing category of maintaining video quality (Category 4). Finally, the root mean square (RMS) method is adapted to dealing with unresolved categories.

#### 3.1.1. Server Modules

The server’s implementation procedure involves monitoring client feedback and assigning the appropriate parameter values to the ongoing video session. It consists of two main modules:

- The H.264 codec with VLCJ media framework captures the video and streams continuously through the HTTP port.
- The second module deals with listening to client feedback messages to adjust the video stream parameter, which includes resolution and frame rate.

#### 3.1.2. Client Modules

The client periodically samples and analyzes the streaming data to estimate the dynamically changing bit rate trend. The client side implementation has three main modules:

- The first module consists of the VLCJ framework for playing streamed media data.
- The implementation of module 2 forms the core of the proposed system, which estimates the client’s bit rate by applying a suitable bit pattern matching algorithm.
- The third module formats the feedback messages in a standard format than can be understood by the server.

### 3.2. Methodology

The proposed system at the client samples the incoming bit rate periodically at $\left(x_{1},x_{2},x_{3},\ldots ,x_{n}\right)$ and analyzes to find the trend of fluctuating data rate during the streaming, as shown in Algorithm 1. The fluctuating bit rate is categorized into predefined patterns (Figure 5) to simplify the estimation process. The proposed algorithm uses the theory of local maxima–minima in sampled bit rate to map the data arrival pattern into one of the four cases: progressive, stabilized, fluctuating, and degraded. When the system cannot resolve the streamed data into any four of these patterns, the status is declared as non-monotonic, and the system computes the data sample’s RMS. By default, the system employs the RMS approach, which includes special cases such as monotonic flat patterns. Predicting the pattern depends on the values of α,β and γ, which are calculated based on analysis of startup, median, and endp for $\left(x_{1},x_{2},x_{3},\ldots ,x_{n}\right)$.
**Algorithm 1.** Client algorithm.(1)Sample the data rate and put it in an array;(2)$\mathrm{Find}\;\mathrm{maxima}\;L_{max}$:(i)$\mathrm{Input}\;\mathrm{bit}\;\mathrm{rate}\;\mathrm{samples}\;\mathrm{in}\;\mathrm{pairs}\;\mathrm{of}\;\mathrm{three}\;\left(v_{1},\;v_{2},\;v_{3}\right)$;(ii)$\mathrm{If}\;\left(v_{1}<v_{2}>v_{3}\right)$, add $v_{2}$ to $L_{max}$, # find maximum bit rate;(iii)Continue step 2 until N received frames;(3)$\mathrm{Find}\;\mathrm{minima}\;L_{\min}$:(i)$\mathrm{Input}\;\mathrm{bit}\;\mathrm{rate}\;\mathrm{samples}\;\mathrm{in}\;\mathrm{pairs}\;\mathrm{of}\;\mathrm{three}\;\left(v_{1},\;v_{2},\;v_{3}\right)$;(ii)$\mathrm{If}\;\left(v_{1}>v_{2}<v_{3}\right)$, add $v_{2}$ to $L_{\min}$, # find minimum bit rate;(iii)Continue step 3 until N received frames;(4)$Max=Analyse\left(L_{max}\right)$, # Call procedure to acquire α, β, and γ;(5)$Min=Analyse\left(L_{min}\right)$, # Call procedure to acquire α, β, and γ;(6)Find status:(2) (i)If (Max==β & & Min==β), set Status as **Progressive**;(ii)Else if (Max==α & & Min==β), set Status as **Stabilized**;(iii)Else if (Max==β & & Min==α), set Status as **Fluctuated**;(iv)Else if (Max==α & & Min==α), set Status as **Degraded**;(v)If (Max==γ || Min==γ),
set Status as **non-monotonic** and call ***Find_rms* (*Bit rates*)**; *A. function Analyze (Bit rates):* Read start data point, *startp;* Read end data point, *endp*; Find median value, *medianp;* If *(startp, medianp, endp)* tends to **monotonic increase**, return β; Else if *(startp, medianp, endp)* tends to **monotonic decrease**, return α; If *(startp, medianp and endp)* tends to neither **monotonic increase** nor **decrease**, return γ; *B. function Find_RMS (Bit rate):* Calculate the root mean square (RMS) of the samples; $X_{rms}=\sqrt{1/n(x_{1}^{2}+x_{2}^{2}+\ldots +x_{n}^{2})}$
Divide the *N* different samples into *M* segments (*M* = 3); Continue Step1 to find the *RMS* values of each segments: ${\mathrm{rms}}_{1}{,\mathrm{rms}}_{2}{,\;\mathrm{and}\;\mathrm{rms}}_{3}$; Compute the difference among the overall RMS and the RMS of the corresponding segments; Calculate $diff_{1}=RMS-rms_{1}$; Calculate $diff_{2}=RMS-rms_{2}$; Calculate $diff_{3}=RMS-rms_{3\;}$; *If*
$\left(diff_{1}<=diff_{2}<=diff_{3}\right)$, then return *1*; *Else if*
$\left(diff_{1}>=diff_{2}>=diff_{3}\right)$, then return *0*; *Else* return 2.

The server side algorithm (Algorithm 2) decodes the client message and modifies the streaming video parameter accordingly. The execution time of the switching process from switching the current stream to the new stream is taken as one input parameter in the server’s decision-making. If the client’s algorithm wrongly classifies the arrival pattern (Ghuge, C. A et al., 2018), then it will lead to an improper action by the server, which may degrade the streaming video on the client side.
**Algorithm 2.** Server side algorithm.Let S
*and*
T be the spatial and temporal resolution vector, respectively, given by: $S=\{SR_{1},\;SR_{2},\;SR_{3},\;SR_{4,}SR_{5}\}$, $T=\{TR_{1},\;TR_{2},\;TR_{3},\;TR_{4},\;TR_{5}\}$.
(1)$\mathrm{Initially}\;\mathrm{set}\;SR_{1}$ and $TR_{1}$ to *a* default value $\left(S_{\mathrm{QCIF}},T_{d}\right)$;
(2)Continue:
Read feedback message (status) from the client; *If*
(Status ==Stabilized),     continue with the existing setup; *Else if* (Status ==Progressive),     call A-LSTM(status), ##Increase Spatial/Temporal resolution; *Else if* (Status ==Fluctuated),     find Switch_time (bit rate),   call A-LSTM(status), ##Update Spatial/Temporal resolution; *Else if* (Status ==Degraded),     call A-LSTM(status), ##decrease Spatial/Temporal resolution; *Else if*
(Status ==Non−monotonic),     wait till next feedback message arrives;
(3)Continue till connection is terminated;
A. *Function Find_Switch_Time (bit rate):* Calculate time for quality switch $T_{switch}=TQS_{k+1}-TQS_{k}$:      #where $TQS_{k+1}$ is the time instant at the end of $k^{th}$ served quality switching,      # request, and $TQS_{k}$ is the present time instant attending previous quality,      # switching request; Set a timer when request for quality switch is received;  Wait for the next feedback message; 
*If*
$\left(T_{switch}>{\;T}_{Fluctuation\_time}\right)$,     discard the request for quality switching and wait for next client      feedback message; *Else*,     serve the request for quality switching.

In this paper, we model the future bit-rate prediction for higher QoS as a time series prediction problem. Time series analysis, which involves analyzing past examples of bit rate in various network qualities to infer an optimal QoS, may be utilized to predict video bit rate. This time series analysis problem is learned in this work using attention-based LSTM (A-LSTM), an advanced variant of recurrent deep neural networks. The A-LSTM algorithm, which was trained using the back propagation through time (BPTT) algorithm, is more useful for learning long-duration dependencies.

**Figure 6:**
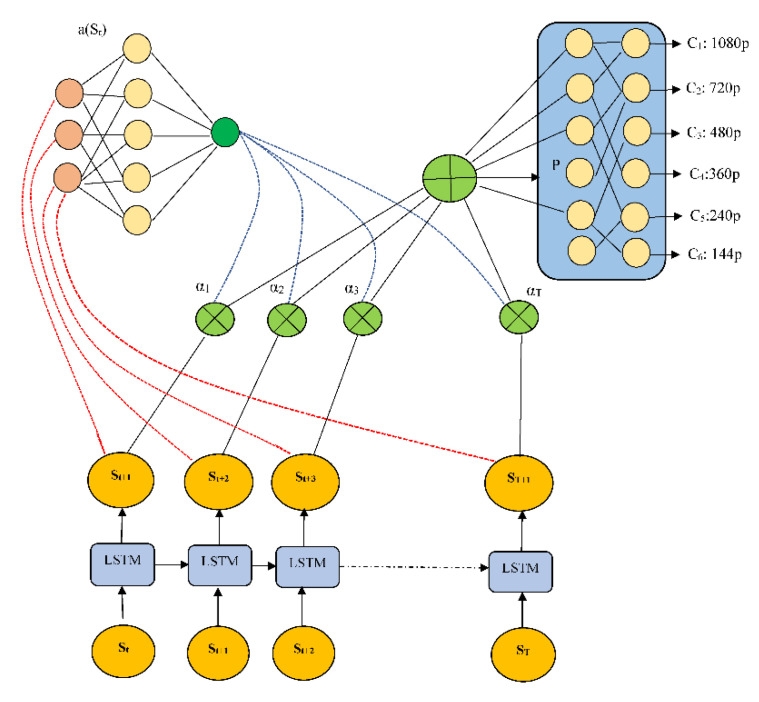
Architecture of the proposed attention based on LSTM at the server module.

To improve the quality of service (QoS) for individual consumers, we use a deep neural model consisting primarily of attention-based LSTM and reinforcement learning architecture (as shown in Figure 6). In reinforcement learning, an agent executes a task on an environment, and the environment responds with a reward based on the action performed. When the LSTM network’s reinforcement learning (RL) agent receives the input state, it chooses an action that is equivalent to the bit rate of the next video sequence. The domain expert examines the performance of the proposed A-LSTM model using the reward function mentioned in Equation (2) based on the action (at) taken. The main goal of the proposed A-LSTM model is to choose an action class for the input state (*S_t_*) that maximizes the overall video quality viewed by the end user. In Equation (1), cost function Q(t) has now been created to assess the total effectiveness of a video streaming session:

(1) $$Q(t)=\sum_{n=1}^{N}q(R_{n})-\mu \sum_{n=1}^{N}T_{n}$$

(2) $$S_{t}=LSTM(S_{t-1})$$

(3) $$e_{t}=a(S_{t})$$

(4) $$\alpha_{t}=\frac{\exp(e_{t})}{\sum_{k=1}^{T}\exp(e_{k})}$$

(5) $$c=\sum_{t=1}^{T}\alpha_{t}S_{t}$$

where the first term $q(R_{n})$ represents the video quality perceived by a user for N video sequence, $S_{t}$ represents the output state, $e_{t}$ represents the feed-forward attention network, $\alpha_{t}$ represents weighted attention, and c represents the weighted feature output.

The inputs to the proposed algorithm are bit rate b_t_, current buffer size as bu_t_, and output of the classification labels related to different spatial resolutions (144p, 240p, 360p, 480p, 720p, and 1080p). Furthermore, during training, attention networks are trained for parameters ′θ′ with the help of rewards given by client feedback messages. Finally, these attention networks are responsible for maintaining efficient adaptive bit rate strategies for a particular video sequence.

The experiment uses the proposed attention-based model, which takes into account the video sequence, download times, and past (k = 16) bit rate measurements. As shown in Figure 6, an LSTM network receives these sequential inputs. The current buffer size as bt, the remaining video sequence as C_t_, and the last chunk bit rate as b_t_, are instantaneous inputs that are fed to a fully connected layer with 128 filters, each of size 4 and stride 1. The final layer, which is fully connected, chooses the state policies’ action for state S_t_ using a softmax function. The softmax function’s output is a selection of the bit rate for the following video segment with the highest probability, ensuring that the best bit rate is chosen for a corresponding state S_t_. In the training phase, user feedback messages are reinforced to the attention network and LSTM network to obtain optimum parameters, using policy gradient strategy. Only the attention-LSTM network is used in predicting spatial resolutions in the testing phase.

For comparison purposes, we implemented a buffer filling algorithm based on the traditional adaptive streaming method [27] to analyze the performance of the proposed A-LSTM system. Implementation of the system at the client level involves monitoring the lower as well as the upper threshold of the media buffer. If the buffer reaches the upper threshold, it recommends slowing down the flow rate, but on the other hand, if the content arrival rate nears the lower threshold, it signals the server to increase the rate of content transfer. As a result, the server reduces or increases the stream bit rate by modifying the resolution of streaming video and/or reducing/increasing frames accordingly.

## 4. Results and Discussion

### 4.1. System Analysis

The streaming content can be treated as a chronological sequence of statistical data, which is sampled periodically for analysis and prediction. The error due to sampling and subsequent analysis need to be formulated and modeled to define the system’s design objective and performance evaluation. The non-parametric approach [28] could be a better approach in micro-level system implementation.

A non-parametric prediction interval can be defined to include a simple maximum and minimum value in a sample set of a given population. Generally, for an exchangeable sequence of random variables, each sample qualifies as the maximum or minimum. In a bit rate sample set of $\left\{R_{0},.\;.\;.\;,R_{n}\right\}$, a sample $R_{i\;}\left(i=0,1,\;\ldots n\right)\;$ has the probability of 1/(n+1)   being the maximum value, and probability of 1/(n+1)   being the minimum value, while (n–1)/(n+1)  of probability, the sample $\;R_{i}$ falls between the largest and smallest sample of $\left\{R_{0},.\;.\;.\;,R_{n}\right\}$. A sample maximum and minimum can be represented by $L_{max}$ and $L_{min}$, respectively, and (n–1)/(n+1) prediction interval of $\left[L_{max}{}_{,}L_{min}\right]$.

For a given sample $R_{i}$, the error of the estimator $\hat{\theta }(R_{i})$ can be denoted as:

(6) $$e\left(R_{i}\right)=\hat{\theta }(R_{i})-\theta_{i}$$

where $\theta_{i}$ is the parameter of estimation. Here, the error $e(R_{i})$ depends on the process of estimation as well as on the sample value. The sampling deviation of the estimator $\hat{\theta }$ for a given sample $R_{i}$, is expressed as:

(7) $$\begin{array}{c} d\left(R_{i}\right)=\hat{\theta }\left(R_{i}\right)-E\left(\hat{\theta }\left(R_{i}\right)\right)\\ =\hat{\theta }\left(R_{i}\right)-E(\hat{\theta }) \end{array}$$

where $E\left(\hat{\theta }\left(R_{i}\right)\right)$ is the expected value of the estimator. Like error of estimator, the sampling deviation $R_{i}$ depends on the estimator as well the sample itself. The variance of $\hat{\theta }$ is computed as the expected value of the square of sampling deviations given by:

(8) $$var(\hat{\theta })=E[{\left(\hat{\theta }-E(\hat{\theta })\right)}^{2}]$$

The variance of the estimate indicates the distance from the expected value of the estimates. Sometimes, the distance between the average of the collection of estimates and the single parameter being estimated, called bias, need to be computed. The bias of *θ* can be denoted as:

(9) $$bia(\hat{\theta })=E(\hat{\theta })-\theta$$

Further,

(10) $$E(\hat{\theta })-\theta =E(\hat{\theta })$$

The mean squared error (MSE) can be expressed in terms of variance and bias as:

(11) $$MSE(\hat{\theta })=\mathrm{var}(\hat{\theta })+{\left(bias(\hat{\theta })\right)}^{2}$$

If $\hat{R}$ denotes a set of *n* predicted values, and R the set of experiential values given as the input to the predictions, then the MSE of the predictor is computed as:

(12) $$MSE=\frac{1}{n}{\sum_{i=1}^{n}\left({\hat{R}}_{i}-R_{i}\right)}^{2}$$

Since the full reference (FR) methods correspond to the objective evaluation of video quality and provide the most accurate result, it was used to evaluate the performance of the proposed system. The two widely used FR metrics are PSNR and SSIM. The paramount interest of the proposed solution is that the system response to the changing network resource should result in higher PSNR and SSIM while sustaining video communication.

#### 4.1.1. Peak Signal-to-Noise Ratio (PSNR)

The PSNR provides information about the degradation of decoded video quality with respect to the original content. It is calculated on luminance components of the video (ITU-T recommendation), which can be formulated on a logarithmic scale as:

(13) $$PSNR=20\;\log_{10}\left(\frac{Max}{\sqrt{MSE(m)}}\right)$$

where $\;Max=2^{no.\;of\;\mathrm{bits}/\left(sample-1\right)}$, and for 8-bit word length, the luminance per sample is 255. The mean squared error (*MSE* (m)) is computed as the absolute difference between the original and the decoded video in the same frame (*m*th), denoted as:

(14) $$MSE(m)=\frac{1}{M\times N}\sum_{i=1}^{M}\sum_{j=1}^{N}{\left[X_{out}(i,j,m)-X_{in}(i,j,m)\right]}^{2}\;$$

The PSNR observation is basically an offline process that can be carried out on a few selected frames at the end of the experiment to ascertain the quality of the streaming system. In designing and developing a higher quality streaming system, achieving a minimum average PSNR of 30 dB may be desirable.

#### 4.1.2. Structural Similarity (SSIM) Index

The SSIM [29] metrics measure the perceived degradation resulting from structural deformation at the frame level. In the real-world video, pixel positions exhibit temporal and spatial dependence between pixels. The spatial dependence information in a frame helps in estimating the structural similarity of the objects in decoded frames; therefore, SSIM is used as a perceptual measure of video quality.

The SSIM [30,31] metric is computed on three different components: luminance, contrast, and structure. It is defined by the Joint Video Team (JVT) of ISO/IEC MPEG and ITU-T VCEG as:

(15) $$l(x,y)=\frac{2\mu_{x}\mu_{y}+C_{1}}{\mu_{x}^{2}+\mu_{y}^{2}+C_{1}}$$

(16) $$c(x,y)=\frac{2\sigma_{x}\sigma_{y}+C_{2}}{\sigma_{x}^{2}+\sigma_{y}^{2}+C_{2}}$$

(17) $$s(x,y)=\frac{\sigma_{xy}+C_{3}}{\sigma_{x}\sigma_{y}+C_{3}}$$

where ${µ}_{x}$ is the average of **x**, ${µ}_{y}$ is the average of **y**, $\sigma_{x}^{2}$ is the variance of **x**, $\sigma_{y}^{2}$ is the variance of **y**, $\sigma_{xy}$ is the covariance of **x** and **y**. The constants $C_{1},C_{2}\;\mathrm{and}\;\;C_{3}$ given by $C_{1}={\left(K_{1\;}L\right)}^{2},\;C_{2}={\left(K_{2\;}L\right)}^{2}and\;C_{3}={\left(K_{3\;}L\right)}^{2}$ are used to stabilize the division operation while dealing with the weak denominator. *L* represents the dynamic range of the pixel values given by:

$L=2^{no.\;of\;bit/pixel}-1$ and $K_{1}<<1$ and $K_{2}<<1$ are two scalar constants.

Using these components, the SSIM is represented as:

(18) $$SSIM(x,y)={\left[l(x,y)\right]}^{\alpha }\cdot {\left[c(x,y)\right]}^{\beta }\cdot {\left[s(x,y)\right]}^{\gamma }$$

where α, β, and γ state the different weightage assigned to each measure. The single-scale SSIM (Yue Wang et al., 2012) is now formulated as:

(19) $$SSIM\left(x,y\right)=\frac{\left(2\mu_{x}\mu_{y}+c_{1}\right)\left(2\sigma_{xy}+C_{2}\right)}{\left(\mu_{x}^{2}+\mu_{y}^{2}+C_{1}\right)\left(\sigma_{x}^{2}+\sigma_{y}^{2}+C_{2}\right)}\;$$

An SSIM index of more than 0.95 represents a good decoded video, and it could be a design objective considering the requirement of the end user in high-quality video communication.

### 4.2. Experimental Setup

The proposed system was implemented in a client–server environment, where the server uses four standard video formats, namely common intermediate format (CIF), quarter CIF (QCIF), sub quarter CIF (SQCIF), and quarter Video Graphics Array (QVGA), to map the video quality dynamically corresponding to the feedback messages. The frame rate of 10, 18, 25, 30, and 35 fps was used to alter temporal resolution. The default value of the temporal resolution (in fps), and also during the initial setup, was assigned as 30. The streaming system at the server selects either one or a combination of spatial and temporal resolutions to achieve the predicted bit rate of the network by the client.

In the experimental setup, the wireless Internet connectivity was established by a 4G Internet-connect device, Reliance_Netconnect+, working on CDMA20001xRTT and 1xEV-DO Rev-A techniques. As per the specification provided by Reliance communication Ltd., the Reliance_Netconnect + device is designed to support a download and upload data rate of 3.1 Mbps and 1.8 Mbps, respectively, but a real-time measurement carried out using an online tool (SpeedOf.Me) had the average bit rate during uplink and downlink estimated at 0.54 Mbps and 0.45 Mbps, respectively, in the laboratory environment. Therefore, fluctuating throughput in data communication across the Internet connected by Reliance_Netconnect + provided us with the ideal real-time platform to test our proposed system.

The different modules of client and server were developed on Dell Inspiron N5010 personal computers having the Intel^®^ Core ™ i7-3770 CPU @ 3.4 GHz processor and 8 GB RAM. The Window-7 Professional 32-bit operating system was installed to run the program for the client/server system. The streaming process was implemented over HTTP with the user datagram protocol as the transport protocol.

**Figure 7:**
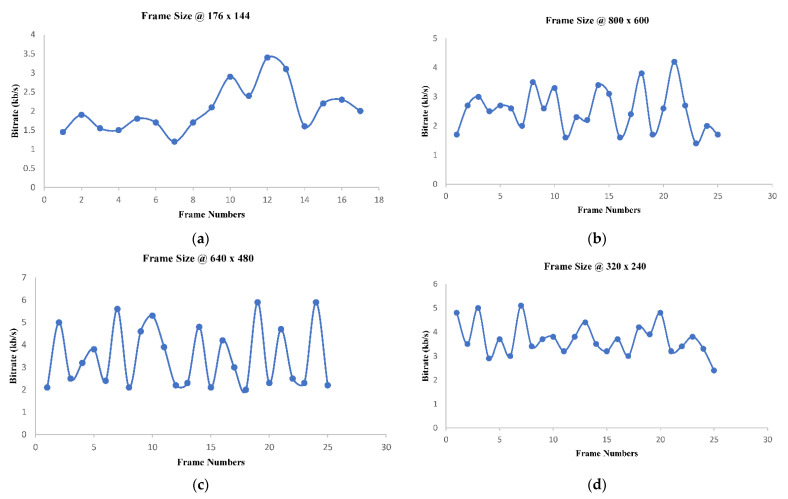
(**a**) Top-Left: 176 x 144, (**b**) Top-Right: 800 × 600, (**c**) Bottom-Left: 640 × 480, (**d**) Bottom-Right: 320 × 240.

H.264 codec generates a variable bit rate for the input video depending on the scene change in visual contents. Figure 7 shows the bit rate vs. frame number for different frame size (176 × 144, 320 × 240, 640 × 480, and 800 × 600) observed during experimentation. The fluctuation in bit rate can be easily observed for all video resolution. This nature of video places additional constraints on system design, as the system proposed is based on best effort service model of the Internet, which operates in the wireless environment.

VLC media player is used in a Java framework (VLCJ) at the sender as well as the receiver. VLCJ provides a higher level framework to cope with the complexities of VLC libraries. The VLC framework includes a variety of media formats through libavcodec library of codecs to the media player, which seamlessly plays the H.264 bit stream. Furthermore, JPCAP provides a library for packet capturing in-network applications using Java, which helps analyze real-time network traffic.

### 4.3. Results and Discussion

The proposed system was implemented using the open-source tool (VLCJ framework), and experimental results were obtained on live as well as stored video in wireless 4G CDMA networks. Since the intended application of this system is to support live video streaming over wireless on the top of HTTP, the result presented here corresponds to live-streamed video in the laboratory environment. Although the working of the system was successfully demonstrated many times, the numerical result analyzed here represents a single instance of experimentation.

#### 4.3.1. Inter-Packet Arrival Delay

**Figure 8:**
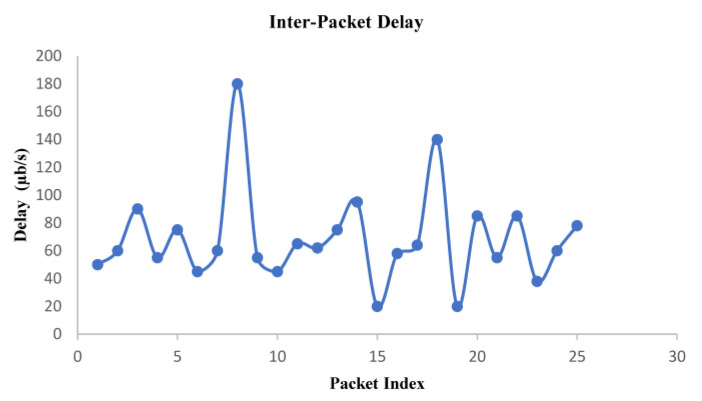
Observed packet arrival delay at the client with a graph drawn between delay and packet index.

The variability of packet delay in the one-way end-to-end communication was observed while discarding packet loss during the experimentation of the proposed video streaming system. As shown in Figure 8, variation in the delay of packet arrival characterizes the inherent property of Internet traffic, which is attributed to the prevailing Internet traffic during the test. The measurement process includes additional delay in 4G wireless, which is used here as last-mile connectivity to the end user. Although the upper bound on packet delay as a design parameter was not directly incorporated in the proposed system, the server dynamically attuned the streaming bit rate to maximize visual eminence. The observed value, plotted in Figure 8, corresponds to an average inter-packet delay of 69 µs.

#### 4.3.2. PSNR Measurement

**Figure 9:**
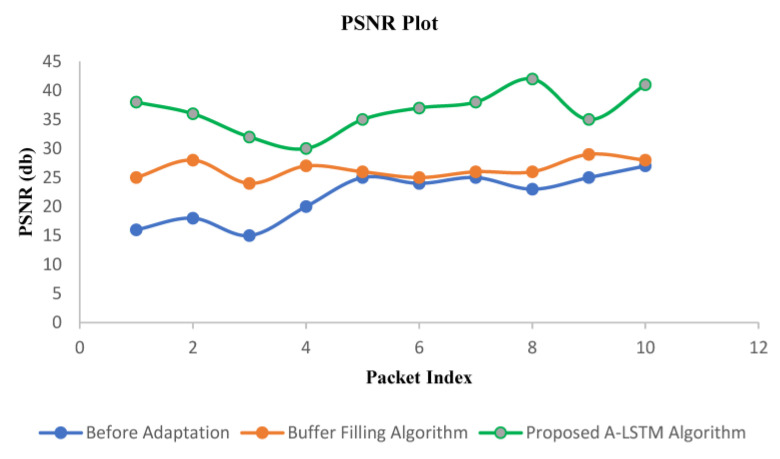
Performance comparison between before adaptation buffer filling algorithm and proposed A-LSTM technique using PSNR value.

The PSNR observation (Figure 9) was performed offline based three methods: (i) without any adaptation, i.e., default existing mechanism in media streaming over the Internet, (ii) buffer filling algorithm, and (iii) the proposed adaptation method. The proposed algorithm achieves an average PSNR of 36.267 dB on video frames resulting from the live streaming, which is 37.53% higher than the buffer-filling algorithm. Further, the average improvement of PSNR is 74.37% higher than the default without an adaptation scheme. The augmented PSNR is accredited to the higher level of adaptation exhibited by the proposed technique, which continuously tries to deliver content at the maximum achievable quality. The observed PSNR for a few frames under different algorithms is also presented in Table 2.

#### 4.3.3. SSIM Index

**Figure 10:**
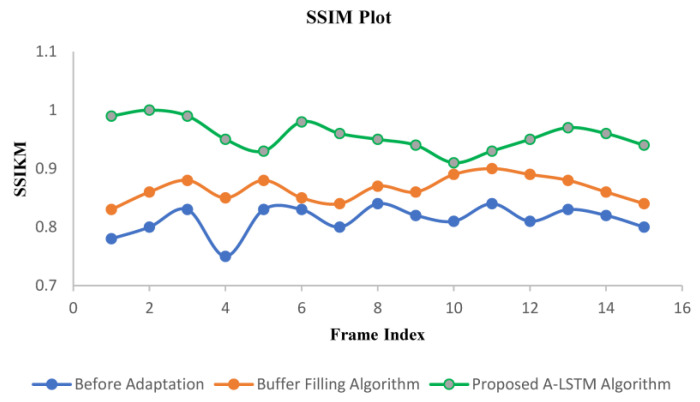
Performance comparison of before adaption, buffer filling algorithm, and proposed A-LSTM algorithm using SSIM index measurement.

The SSIM was calculated offline using the same approach as that of PSNR. It is observed based on the data generated by three methods: default without adaptation scheme, existing buffer filling algorithm, and proposed adaptation algorithm resulting from the live streaming of video (Figure 10). The system implementing the proposed algorithm provides a 5.7% increase in average SSIM index than the existing buffer filling algorithm, and it achieves a much higher (11.44%) index than the method without adaptation. Due to the higher level of adaptation exhibited by the proposed system, the structural statistics were preserved, resulting in a higher value of the SSIM index. Although the design and implementation of the system do not directly deal with the retention of structural property during streaming, a higher SSIM index is an incentive for the system dynamics. Table 3 lists the perceived numerical ethics of SSIM under different adaptation algorithms.

#### 4.3.4. Selected Original and Decoded Frame

**Figure 11:**
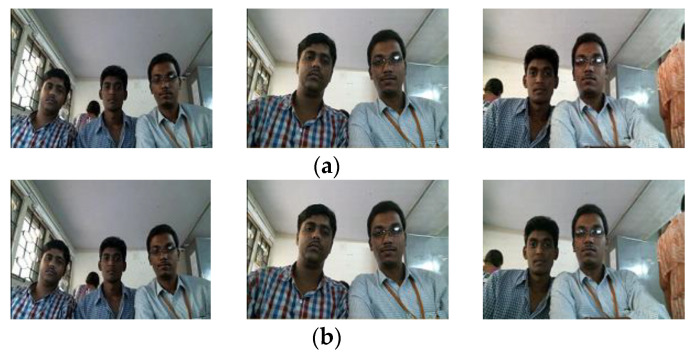
Quality comparison of sampled frames between (**a**) Original and (**b**) Received online video frames.

**Figure 12:**
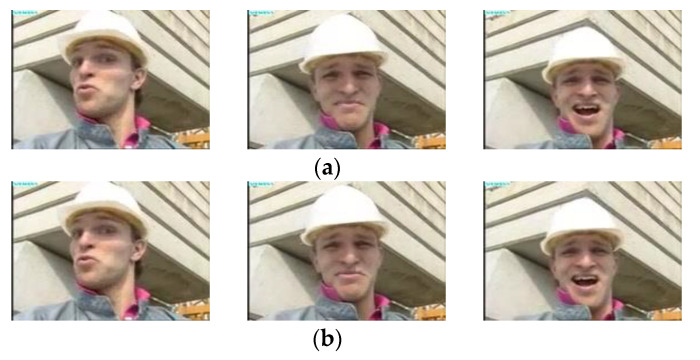
Quality comparison between (**a**) Original and (**b**) Received sampled stored foreman video frames.

Figure 11 and Figure 12 show the original and received decoded frames recorded during live streaming and stored foreman video during experimentation. Since the proposed system maintains an average PSNR of more than 36 dB and an SSIM index of 0.96, even a keen look at the received frames does not reveal a noticeable loss in quality of the decoded video, which is a requirement in developing a system for high-quality video applications. This also emphasizes the proposed method’s importance over existing approaches and the default mechanism available on the Internet.

## 5. Conclusions

The development of a mechanism to support adaptive streaming of video over HTTP in dealing with fluctuation in available bit rate on the Internet with last-mile connectivity as a wireless network is a rewarding approach. The proposed A-LSTM system adopted the theory of maxima–minima along with an RMS method, reinforcement techniques, along with attention-LSTM networks to compute and match the pattern of the bit stream. It also included the duration of network fluctuation as well as the time for switching excellence in effective decision-making, while switching between different video qualities. The proposed solution tackled the problem of inherent conduct of wireless and Internet traffic in a unified tactic. The link level quality of service parameters such as delay, jitter, and packet loss was not considered in the problem formulation as the system is developed on top of HTTP. 

Although the proposed system is targeted toward the attainment of quality of the video, it can be used in many other video streaming applications. Abrupt congestion at the router on the Internet may cause extra delay in video streaming packets, and a suitable method is required to tackle it. Another future work includes supporting video streaming to hand-held devices (smart phones) connected to the Internet through a cellular network.

## Figures and Tables

**Table 1:** Test factors as per the ITU-T J.247 recommendation.

S. No.	Parameters	Values
1	Transmission	Errors with packet loss
2	Frame rate	5 fps to 30 fps
3	Video codec	H.264/AVC, VC-1, Windows Media 9, Real Video, MPEG4
4	Video resolution (QCIF, CIF, and VGA)	QCIF (6 to 320 Kbps) CIF (64 to 2000 Kbps) VGA (128–4000 Kbps)
5	Temporal errors (pausing with skipping)	Maximum of 2 s

**Table 2:** PSNR values for different frames.

Frame Number	Before Adaptation	Buffer Filling Algorithm	Proposed A-LSTM Algorithm
1	19.54	25.89	39.09
2	19.83	26.24	39.09
3	18.47	24.89	36.79
4	20.14	28.64	32.69
5	23.54	27.59	34.9
6	22.58	26.49	33.98
7	21.29	25.87	35.01
8	20.48	25.98	39.09
9	20.89	26.87	34.32
10	21.23	25.23	37.71
**Average**	**20.799**	**26.369**	**36.267**

**Table 3:** SSIM values for different frames.

Frame Number	Before Adaptation	Buffer Filling Algorithm	Proposed A-LSTM Algorithm
1	0.835	0.873	0.965
2	0.863	0.892	0.986
3	0.768	0.912	0.967
4	0.887	0.925	0.956
5	0.89	0.899	0.978
6	0.884	0.924	0.968
7	0.869	0.934	0.946
8	0.894	0.939	0.952
9	0.873	0.934	0.968
10	0.879	0.916	0.973
**Average**	**0.8689**	**0.9160**	**0.9683**

## Data Availability

The data used to support the findings of this study will be available with the corresponding author.
